# 2D:4D Ratio in Neurodevelopmental Disorders: A Twin Study

**DOI:** 10.1007/s10803-018-3588-8

**Published:** 2018-04-27

**Authors:** Lynnea Myers, Annelies van’t Westeinde, Ralf Kuja-Halkola, Kristiina Tammimies, Sven Bölte

**Affiliations:** 1 Division of Neuropsychiatry, Department of Women’s and Children’s Health, Center of Neurodevelopmental Disorders (KIND), Karolinska Institutet & Center for Psychiatry Research, Stockholm County Council, Stockholm, Sweden; 2 Department of Medical Epidemiology and Biostatistics, Karolinska Institutet, Stockholm, Sweden; 3 Division of Neuropsychiatry, Department of Women’s and Children’s Health, Center of Neurodevelopmental Disorders (KIND), Karolinska Institutet & Child and Adolescent Psychiatry, Center for Psychiatry Research, Stockholm County Council, Gävlegatan 22B, 113 30 Stockholm, Sweden

**Keywords:** Autism, ADHD, 2D:4D ratio, Neurodevelopmental disorders, Sex, Hormones, Twins

## Abstract

The second to fourth digit (2D:4D) ratio is of interest in autism spectrum disorder (ASD). Studies on the relationship of this ratio with other neurodevelopmental disorders (NDDs) are lacking. Investigating the association between the ratio and NDDs in twins can provide insight into genetic and/or environmental factors driving the ratio. Hand images were collected in *N* = 238 twins with NDDs or typical development from 70 monozygotic and 49 dizygotic pairs to examine ratios and their associations to DSM-5 defined categorical NDDs, autistic traits, zygosity, and sex. There were small associations for males between the ratios and any NDD and ADHD diagnoses. Males had lower ratios than females. Future studies exploring the ratio alongside physical anomalies could provide etiological insight into NDDs.

**Electronic supplementary material:**

The online version of this article (10.1007/s10803-018-3588-8) contains supplementary material, which is available to authorized users.

## Introduction

The ratio of the second to fourth finger digits (hereafter referred to as “2D:4D ratio” or just “ratio”) refers to the length of the second finger digit (index finger) divided by the length of the fourth finger digit (ring finger). The ratio has been suggested to serve as a biomarker for prenatal androgen activity for a variety of conditions, including autism spectrum disorder (ASD), congenital adrenal hyperplasia, and Klinefelter syndrome (Manning et al. 2014, 1998). Indeed, Lutchmaya et al. (2004) explored the 2D:4D ratio in human fetuses and found that higher levels of testosterone compared with estrogen were associated with lower ratios, while lower levels of testosterone compared with estrogen were associated with higher ratios. Since low 2D:4D ratios are related to higher fetal testosterone and lower fetal estrogen exposure, lower ratios are more common in males and higher ratios in females (Galis et al. 2010; Hampson et al. 2008; Malas et al. 2006; Manning et al. 2002, 1998, 2004; Voracek and Dressler 2007).

The 2D:4D ratio in relationship to ASD has been examined, in part, due to the extreme male brain theory (Baron-Cohen et al. 2005), which purports an influence of fetal testosterone exposure on the development of ASD. The majority of the studies have shown lower 2D:4D ratios in individuals with ASD (Al-Zaid et al. 2015; de Bruin et al. 2009; Honekopp 2012; Manning et al. 2001; Teatero and Netley 2013), although a recent study with nearly 6000 children failed to find a relationship between a lower ratio and ASD or autistic traits (Guyatt et al. 2015). Mixed results have been shown for the association of the ratio with autism symptoms, autistic traits, as well as other autism related endophenotypes. de Bruin et al. (2009) explored the 2D:4D ratio in ASD, other neurodevelopmental disorders (NDDs) and psychiatric disorders and found a negative association for the left-hand ratio with autism symptoms on the Autism Diagnostic Observation Schedule—Generic in a small subsample of girls. Empathizing traits (assumed to be higher in females) and systemizing traits (assumed to be higher in males) have been explored previously in studies on ASD, but a review by Honekopp (2012) on the association between the digit ratio these traits found no evidence for a link in typically developing adults, which is in alignment to earlier findings by Voracek and Dressler (2006).

Although the sex ratio is skewed in most NDDs (American Psychiatric Association 2013), the exploration of the 2D:4D ratio is limited in other NDDs, such as attention-deficit/hyperactivity disorder (ADHD), intellectual disability (ID), communication disorders, specific learning disorders, and motor disorders or broader defined psychiatric groups. However, one study (de Bruin et al. 2006) showed that males with ASD and ADHD had lower 2D:4D ratios in comparison with males with anxiety disorders or typical development.

A previous study investigating the 2D:4D ratio in twins suggests that the ratio is highly heritable, with an estimate around 80% (Voracek and Dressler 2007). The study included typically developing twins only, not clinical samples with NDDs. Twin studies provide insight into the proportion of genetic and environmental factors influencing phenotypes like the ratio. The main premise of the twin design is that monozygotic twins share nearly identical genetic information and therefore, differences in outcomes like the ratio can be attributed with high likelihood to environmental factors (Martin et al. 1997; Willfors et al. 2017).

Technically, studies exploring digit ratios have primarily used either scanned or photocopied images of the palmar surface of hands, along with calipers, to indirectly measure digit length (Al-Zaid et al. 2015; de Bruin et al. 2009; Guyatt et al. 2015; Manning et al. 2001). Indirect measurements using photocopies of hands have been shown to be highly repeatable, but may result in lower digit ratios compared with direct measurements. This lower ratio is speculated to be due to changes that occur in the fat pad and finger curvature when taking a photocopy of the hand versus direct measurement (Manning et al. 2005). On the contrary, a previous study by Manning et al. (2000) found no difference between direct and indirect measurements and similarly, a meta-analytic review by Honekopp (2012) found the method of measurement of the 2D:4D ratio used in the studies reviewed for individuals with ASD did not have an effect on the ratio. Medical photographs can be taken of hands so that the fingers are not compressed on a photocopier or scanner. Additionally, the use of medical photography allows for easy use of a digital measurement programs to assess digit length. Digital measurement programs are now freely available and can quickly measure finger lengths in images, with the possibility for automation in the future to measure finger lengths in a large number of images quickly and precisely.

Because there is substantial overlap among NDDs and research shows higher rates of ASD and ADHD in males (Ramtekkar et al. 2010; Werling and Geschwind 2013), further studies are desirable to examine the association between NDDs, neurodevelopmental traits, and the ratio across sexes. Furthermore, to the authors’ best knowledge, no study has yet examined the 2D:4D ratio in a sample of twins with NDDs, which could provide insight into genetic and/or environmental influences on the ratio. Thus, this study sought to investigate the 2D:4D ratio in a carefully characterized, rare sample of monozygotic (MZ) and dizygotic (DZ) twins concordant or discordant for ASD, ADHD and other NDDs as well as typically developing (TD) control pairs using digital measurement of high-quality medical photographs of hands. We aimed to examine the association between the 2D:4D ratio for (i) NDDs as a whole, ASD and ADHD separately, and autistic traits, (ii) sex, and (iii) zygosity. We predicted lower 2D:4D ratios in individuals with categorical diagnoses of NDD and males compared to TD and females, respectively, a negative correlation between the ratio and autistic traits, and higher correlations of the ratio in MZ versus DZ twins.

## Method

### Sample

The study was approved by the Regional Ethical Review Board Stockholm, Sweden and consent was obtained from participants and/or parents prior to the start of the study. Twins were recruited from the Roots of Autism and ADHD Twin Study in Sweden (RATSS), described elsewhere in detail (Bölte et al. 2014), from August 2011 to March 2017. RATSS recruits twins through the Child and Adolescent Twin Study in Sweden (CATSS, Anckarsater et al. 2011), via advertisements in journals of national Swedish NDD interest organizations, referrals from clinical units (e.g., child psychiatry, habilitation centers), and the Swedish patient registry. TD twins were also recruited from the Child and Adolescent Twin Study in Sweden (CATSS, Anckarsäter et al. 2011). The TD twins in our study were randomly sampled from the aforementioned population-based twin study, and although they are assumed to be representative of twins in a population, they also represent individuals interested in participating in a research study.

This study reports on a total of *N* = 238 twins, representing 70 MZ pairs and 49 DZ pairs. Of all twin individuals, 106 (44.5%) had a NDD diagnosis and 132 (55.5%) were TD. Separate NDD diagnoses (numbers unadjusted for NDD comorbidities, so that individuals may have multiple diagnoses) were 46 ASD, 64 ADHD, 11 ID, and 38 other NDDs (e.g., motor, communication, or specific learning disorders). About 55% were male (*n* = 132; 67 with NDDs) and 45% female (*n* = 106; 39 with NDDs). Ages at examination ranged from 8 to 29 years (*M* = 16.2, *SD* = 5.2) (see Table 1).

**Table 1:** Sample characteristics by diagnosis^a^ and concordance for 238 twins (119 pairs)

	TD	ASD			ADHD			NDD		
	Concordant	Concordant	Discordant		Concordant	Discordant		Concordant	Discordant	
Number of pairs	45	9	28		16	32		32	42	
Zygosity by pairs (MZ:DZ)	31:14	7:2	12:15		10:6	11:21		19:13	20:22	
Sex by twin (female:male)	50:40	8:10	20:36		12:20	24:40		22:42	34:50	
Age, mean (SD, range)	18.7 (5.4, 10–29)	14.1 (2.8, 10–17)	15.3 (4.8, 9–28)		13.6 (3.1, 8–18)	13.7 (4.5, 8–28)		13.9 (3.2, 8–19)	15.4 (5.1, 8–28)	

			Affected twin	Co-twin		Affected twin	Co-twin		Affected twin	Co-twin
IQ, mean (SD, range)	101.3 (13.0, 74–131)	93.8 (21.7, 63–142)	97.1 (18.6, 69–138)	101.5 (15.1, 65–123)	90.1 (16.6, 64–121)	101.4 (14.4, 71–127)	101.2 (11.1, 83–123)	93.4 (17.9, 62–142)	97.4 (15.8, 68–127)	100.8 (13.3, 74–123)
SRS-2, total raw mean (SD, range)	23.3 (19.0, 2–89)	76.6 (28.1, 38–128)	81.2 (26.9, 21–130)	34.9 (24.5, 2–93)	73.9 (38.6, 20–142)	59.4 (30.7, 5-117)	29.0 (17.3, 2–79)	64.2 (33.2, 12–142)	59.5 (30.6, 1-117)	23.6 (13.1, 1–54)
# Participants with other diagnoses										
ASD: n (%)		18(100.0)	28 (100.0)	0 (0.0)	11 (34.4)	11 (34.4)	4 (12.5)	26 (40.6)	20 (47.6)	
ADHD: n (%)		10 (55.6)	12 (42.9)	5 (17.9)	32 (100.0)	32 (100.0)	0 (0.0)	40 (62.5)	24 (57.1)	
ID: n (%)		3 (16.7)	3 (10.7)	1 (3.6)	5 (15.6)	0 (0.0)	0 (0.0)	8 (12.5)	3 (7.1)	
Other NDD: n (%)		1 (5.6)	10 (35.7)	4 (14.3)	11 (34.4)	8 (25.0)	4 (12.5)	24 (37.5)	14 (33.3)	

*MZ* monozygotic, *DZ* dizygotic, *SD* standard deviation, *IQ* intelligence quotient, *ASD* autism spectrum disorder, *ADHD* attention-deficit/hyperactivity disorder, *ID* intellectual disability, *NDD* neurodevelopmental disorder

^a^Unadjusted for NDD comorbidities

Power calculations are complicated for the current study, mainly because the analyzed sample is not randomly selected from the source population, but rather sampled to incorporate relatively more MZ pairs and pairs with NDDs. Previous reviews regarding the relationship between the ratio and ASD have found effect sizes ranging from − .43 (Teatero and Netley 2013) to − .58 (Honekopp 2012). Using these reported effect sizes, to find an effect (with equally sized affected and non-affected groups), the sample size needed to have 80% power to detect significant differences between groups at a *p* value of .05 lies between 96 (*d* = 0.56) and 172 (*d* = 0.43) participants. The corresponding sample sizes needed with 90% power are 128 (*d* = 0.56) and 230 (*d* = 0.43). No studies were identified to provide effect sizes for samples with other types of NDDs.

### Diagnostic and Behavioral Assessments

Participants were diagnosed using a consensus process with several experienced clinicians according to DSM-5 criteria based on information from the following standardized instruments: Autism Diagnostic Observation Schedule 2nd Edition (ADOS-2; Lord et al. 2012); Autism Diagnostic Interview-Revised (ADI-R; Rutter et al. 2003); Kiddie Schedule for Affective Disorders and Schizophrenia (K-SADS; Kaufman et al. 1997); and Diagnostic Interview for ADHD in Adults (DIVA 2.0; Kooij 2010). IQ testing was performed with the following measures: Wechsler Adult Intelligence Scale-IV (WAIS-IV; Wechsler 2003a), Wechsler Intelligence Scale for Children-IV (WISC-IV; 2003b); and the Leiter International Performance Scale-Revised (Roid and Miller 1997). Twin pairs were categorized as either NDD-concordant (i.e., both twins meeting criteria for NDD diagnosis), NDD-discordant (i.e., only one twin in pair meeting criteria for NDD diagnosis), or TD (i.e., neither twin meeting criteria for NDD diagnosis). Autistic traits were measured with the Social Responsiveness Scale-2 (SRS-2, Constantino and Gruber 2012). The SRS-2 provides a quantitative measure of autistic traits related to social awareness, cognition, communication, and motivation, as well restricted interests and repetitive behaviors. SRS-2 total raw scores were applied based on recommendations for its use in research settings. Increasing total raw scores (0–195) on the SRS-2 indicate greater autistic traits. Saliva and blood were collected to confirm zygosity through genotyping with Infinium Human-CoreExome chip (Illumina) or using a panel of 47 validated single nucleotide polymorphisms (Hannelius et al. 2007).

### Digit Measurement

Photographs of the right and left hand of each participant were obtained by the medical photography lab at Karolinska University Hospital. The photographs were taken with each participant’s hands lying flat, palmar surface up on a small table covered with a dark cloth. Participant hand images were uploaded into Image J (Schindelin et al. 2015) and the “straight” measurement tool in this program was used to measure each digit on both the right and left hand. The measurement of each digit was taken from the midpoint of the arc defining the tip of each digit vertically to the most proximal crease of the digit in the palm of the hand. Two raters, who were blinded to the participants’ diagnoses, measured the digits on each hand independently and noted those cases for which digit measurements were not usable. Exclusion criteria included the digit not being flat enough on the table surface or the hands or digits being curled or cupped. Participants lacking accurate digit measurements for either the second and/or fourth digit on either the right or left hand were removed from the final sample, along with their co-twin. A total of 16 twin pairs were removed from the final analysis due to issues with digit measurement in one of the twins (25% TD), while three pairs removed due to issues with measurement in both of the twins (33% TD). Excluding individuals with issues that prevent adequate finger measurements is consistent with previous studies on ratios in ASD (Teatero and Netley 2013).

An intraclass correlation coefficient (*ICC*) was calculated to explore agreement between the ratios of the second and fourth digits from both the right hand and left hand between the two raters using a two-way random-effects model with absolute agreement. The *ICC* was *r* = .93 for both the left and right hand, indicating high reliability. Previous studies on the 2D:4D ratio broadly reported mean ratios derived individually from both the right and left hands (e.g., de Bruin et al. 2009; Galis et al. 2010; Lutchmaya et al. 2004; Manning et al. 1998, 2001; Trivers et al. 2006; Voracek and Dressler 2007), the right hand only (e.g., Al-Zaid et al. 2015; Malas et al. 2006; Manning et al. 2004), either the right or left hand (e.g., de Bruin et al. 2006), or a mean that combines measures from both the right and left hands (e.g., Guyatt et al. 2015; Manning et al. 2001; Voracek and Dressler 2007). In the review by Teatero and Netley (2013), the authors suggest the right versus left 2D:4D ratio may be more strongly associated with ASD. In our study of participants with NDDs, in order to examine the association between the right and left hand ratios, a Spearman’s rho correlation was estimated between the right and left hand and was found to be robustly correlated (*r* = .59, *p* < .001), a finding similar to previous studies (Guyatt et al. 2015; Manning et al. 2001). Due to the consistent bilateral correlation between hand ratios, a bilateral combined mean 2D:4D hand ratio was calculated across raters and was used for the primary analyses in the study. Additionally, in accordance with earlier studies exploring the right and/or left hand ratios, ad hoc analyses exploring the relationship between NDD diagnoses and right and left ratios using the between-pairs model were also performed.

### Statistical Analysis

Statistics were calculated with SPSS version 24 and R version 3.3.2. Different sex DZ twin pairs were removed (*n* = 6 pairs, resulting in a total of *n* = 49 DZ pairs after exclusion) due to the potential effect of testosterone from the male twin on the female co-twin’s ratio as discussed in Voracek and Dressler (2007). Since the overall mean 2D:4D ratio was not normally distributed, median and interquartile ranges (*IQR*) are reported for 2D:4D ratios by diagnosis and concordance type, as well as by gender. To adjust for the clustering in pairs, a conditional regression model was fitted using generalized estimating equation (GEE) analyses (Zetterqvist et al. 2016) for assessments of the association between the 2D:4D ratio (predictor) and (i) all NDD diagnoses (including ASD and ADHD), (ii) ASD only, (iii) ADHD only, (iv) other NDDs only (e.g., motor, communication, or specific learning disorders), and (v) autistic traits (outcomes). The association between the ratio and NDDs were examined with two models: first, a conventional linear regression model for estimates of associations between the ratios and NDDs (referred to as *between-pairs estimates)* using clustered standard errors accounting for the twin correlation; and second, a conditional linear regression model for estimates of association within-pairs (referred to as *within-pairs estimates*) after adjusting for factors shared within twins. The within-pairs estimates account for shared factors by investigating whether the twin in a pair that had a lower ratio (compared to his/her co-twin) also an NDD diagnosis. Significance was defined a priori as a *p* value of .05 or less.

A frequently cited study by Trivers et al. (2006) found in a sample of 108 Jamaican children ages 7–17 years that the ratio increased slightly with age, while previous studies by Manning et al. (1998) with 800 participants (ages 2–25 years) and Malas et al. (2006) with 161 human fetuses (9–40 weeks gestation) found the ratio to remain stable with age. Despite the controversy in the literature, age was covaried for in the between-pair analyses with the conventional linear regression model exploring the relationships between the ratio and NDD diagnoses. Age is implicitly controlled for in the within-pair analyses as age for both twins is identical.

Contrary to an earlier study (Luxen and Buunk 2005), there was no association between the ratio and IQ in our sample (*beta* = − .0002, *p* = .164 without adjusting for age, *beta* = − .0002, *p* = .154 with adjusting for age; beta represents how much the ratio is raised or lowered for every one-point increase in IQ). Thus, IQ was not adjusted for in our analyses.

## Results

### 2D:4D Ratio by Sex

The 2D:4D ratio for females was *Md* = 1.000 (*IQR* 0.972 and 1.028) for the right hand, *Md* = 1.014 (*IQR* 0.982 and 1.038) for the left hand, and Md = 1.010 (*IQR* 0.981 and 1.031) for the overall hand. The 2D:4D ratio for males was *Md* = .987 (*IQR* 0.965 and 1.017) for the right hand, Md = 0.989 (*IQR* 0.971 and 1.019) for the left hand, and *Md* = 0.992 (*IQR* 0.970 and 1.015) for the overall hand (for distributions of the overall mean ratios for all participants and split by sex, see the Supplemental Fig. 1a–c). As hypothesized, participant sex predicted the overall hand ratio, with male sex being associated with ratios lowered by .011 (*p* = .044). The results below explore the relationship between the overall hand ratio and the various NDD diagnoses and dimensional traits split by sex.

### 2D:4D Ratio by NDD Diagnoses, Autistic Traits, and Concordance

The 2D:4D ratio was associated with the presence of NDD as a group for males in the between-pairs model (*beta* = − .014, 95% CI − .025 to − .002 *p* = .019) and for females in the within-pairs model (*beta* = − .017, 95% CI − .035 to .000, *p* = .050), indicating that for both males and females in the respective models, the ratio decreased with the presence of any NDD diagnosis. The ratio was also associated with the presence of ADHD for males in the between pairs model (*beta* = − .015, 95% CI − .027 to − .003, *p* = .012). No associations were found for ASD or other NDDs (e.g., motor, communication, or specific learning disorders) as separate NDD subgroups, in either the between- or within-pairs model, including when examined separately by sex or when adjusted for the presence of ASD, ADHD, or any NDD, respectively (see Table 2). Analyzing autistic traits, an association with the ratio was found for females in the between-pairs model (*beta* = .0002, 95% CI .0000–.0003, *p* = .016), indicating that for every point increase on the SRS-2, there was a .0002 increase in the ratio. No associations between autistic traits and the ratio were found for males, nor for either sex in the within-pairs model (see Table 2).

**Table 2:** Between- and within-pairs associations: overall hand 2D:4D ratio, NDD diagnoses, autistic traits and IQ

Categorical or dimensional diagnoses	Between-pairs estimate (95% CI)	Within-pairs estimate (95% CI)	MZ only within-pairs estimate (95% CI)	DZ only within-pairs estimate (95% CI)
Any NDD and ratio				
No adjustments	− .008 (− .018 to .002)	− .008 (− .019 to .004)	− .006 (− .018 to .006)	− .010 (− .028 to .009)
Split by gender (M)	− .014* (− .025 to − .002)	− .002 (− .016 to .013)	− .006 (− .023 to − 011)	.004 (− .020 to .027)
Split by gender (F)	.002 (− .012 to .015)	− .017* (− .035 to .000)	− .006 (− .019 to .007)	− .025 (− .053 to .002)
ASD and ratio				
No adjustments	− .002 (− .014 to .009)	− .008 (− .021 to .006)	− .009 (− .022 to .003)	− .006 (− .029 to .017)
Split by gender (M)	− .008 (− .022 to .006)	− .005 (− .024 to .013)	− .013 (− .033 to .006)	.001 (− .029 to .030)
Split by gender (F)	.006 (− .009 to .021)	− .012 (− .029 to .006)	− .003 (− .011 to .005)	− .020 (− .052 to .012)
Adjusted for ADHD	− .001 (− .012 to .011)	− .008 (− .022 to .005)	− .010 (− .024 to .003)	− .006 (− .030 to .017)
Adjusted for NDDs	− .006 (− .018 to .007)	− .009 (− .024 to .005)	− .010 (− .023 to .003)	− .009 (− .033 to .015)
ADHD and ratio				
No adjustments	− .008 (− .019 to .003)	.001 (− .014 to .016)	.004 (− .013 to .022)	− .001 (− .021 to .019)
Split by gender (M)	− .015* (− .027 to − .003)	.008 (− .010 to .026)	.007 (− .016 to .030)	.009 (− .017 to .035)
Split by gender (F)	.006 (− .014 to .021)	− .011 (− .034 to .012)	− .002 (− .021 to .018)	− .014 (− .045 to .016)
Adjusted for ASD	− .008 (− .019 to .003)	.003 (− .012 to .018)	.006 (− .011 to .024)	.001 (− .021 to .022)
Adjusted for NDDs	− .010 (− .021 to .002)	− .003 (− .017 to .012)	.002 (− .014 to .019)	− .006 (− .028 to .016)
Other NDDs and ratio				
No adjustments	− .003 (− .017 to .010)	− .010 (− .025 to .004)	− .006 (− .022 to .009)	− .013 (− .036 to .010)
Split by Gender (M)	− .005 (− .024 to .013)	− .008 (− .029 to .013)	− .008 (− .027 to .012)	− .009 (− .049 to .032)
Split by Gender (F)	.001 (− .016 to .017)	− .014 (− .030 to .002)	− .002 (− .017 to .013)	− .017 (− .037 to .002)
Adjusted for ASD	− .003 (− .017 to .011)	− .009 (− .023 to .006)	− .004 (− .021 to .014)	− .012 (− .034 to .009)
Adjusted for ADHD	− .002 (− .016 to − 012)	− .011 (− .024 to .003)	− .008 (− .023 to .008)	− .013 (− .034 to .009)
IQ and ratio				
No adjustments	.0003 (− .0008 to .0015)	− .0001 (− .0005 to .0004)	.0004 (− .0001 to .0009)	− .0002 (− .0009 to .0004)
SRS-2 and ratio				
No adjustments	.0001 (− .0001 to .0002)	.0000 (− .0002 to .0002)	.0001 (− .0002 to .0003)	.0000 (− .0003 to .0003)
Split by gender (M)	− .0002 (− .0002 to .0001)	.0001 (− .0001 to .0004)	.0000 (− .0003 to .0003)	.0002 (− .0002 to .0005)
Split by gender (F)	.0002* (.0000 to .0003)	− .0001 (− .0005 to .0003)	.0002 (− .0003 to .0006)	− .0004 (− .0009 to .0001)

Beta represents the amount by which the 2D:4D ratio is lowered by each variable explored. Between-pair estimates are adjusted for age

*Ratio* second digit and fourth digit (2D:4D) ratio, *IQ* intelligence quotient, *ASD* autism spectrum disorder, *ADHD* attention-deficit/hyperactivity disorder, *NDD* neurodevelopmental disorder, *SRS-2* social responsiveness scale-2, *M* male, *F* female

*Associations significant at p < .05

**Fig. 1:**
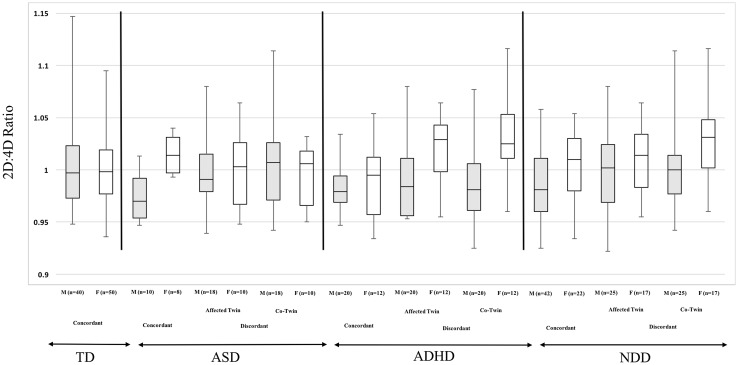
Box plots illustrating the median 2D:4D ratio, as well as 25th and 75th interquartile range and minimum and maximum values by gender based on TD or diagnosis and concordance of diagnosis. Female co-twins (unaffected) discordant for NDDs had the highest median digit ratio (Md = 1.031), followed by affected females twins discordant for ADHD (Md = 1.029). Males with concordant ASD had the lowest ratio (Md = .970) followed by male co-twins discordant for ADHD (Md = .981). *2D:4D* second digit and fourth digit ratio, *TD* typical development, *ASD* autism spectrum disorder, *ADHD* attention-deficit/hyperactivity disorder, *NDD* neurodevelopmental disorder

The pattern of a slightly higher median overall hand ratio for females compared to males was consistent in twin pairs discordant for NDDs, ASD, and ADHD (see Fig. 1). The median hand 2D:4D ratio for males with concordant TD was *Md* = 0.997 (*IQR* 0.973 and 1.023) and for females *Md* = .998 (*IQR* .977 and 1.019). For males with concordant NDD, the ratio was *Md* = 0.981 (*IQR* 0.960 and 1.011) and for females *Md* = 1.010 (*IQR* 0.980 and 1.030). In concordant ASD, the ratio in males was Md = 0.970 (*IQR* 0.954 and 0.992) and in females *Md* = 1.014 (*IQR* 0.997 and 1.031). Finally, the ratio for males with concordant ADHD was *Md* = .979 (*IQR* 0.969 and 0.944) and for females was Md = 0.995 (*IQR* 0.957 and 1.012).

### 2D:4D Ratio by Zygosity

The 2D:4D ratio for the overall hand in MZ pairs was *Md* = 1.001 (*IQR* 0.975 and 1.023) and for DZ pairs was *Md* = 0.993 (*IQR* 0.966 and 1.022). The Spearman’s correlation for the ratio between twins in MZ pairs and DZ pairs, respectively, was significant (MZ: *r*_*s*_ = .670, *p* < .001; DZ: *r*_*s*_ = .363, *p* = .010), although the strength of the association was much higher for MZ pairs, most likely due to the strong heritability of the ratio as reported in previous studies (Voracek and Dressler 2007). When examining the within-pairs estimates of the relationships between the ratio and the various NDDs in MZ compared to DZ pairs, no significant relationships were identified.

## Discussion

This study examined the association between the 2D:4D ratio and NDD diagnoses, as well as autistic traits. Findings replicate previous observations of sexual dimorphism in the ratio with a lower overall 2D:4D ratio in males compared to females (Galis et al. 2010; Malas et al. 2006; Manning et al. 2004). As expected, 2D:4D ratios were highly correlated in MZ twins, who share all or nearly all their genetic information, and also in DZ twins, who share 50% of their genetic profiles. An association was found between the 2D:4D ratio and the presence of any NDD and ADHD diagnoses in males in the between-pairs model and any NDD in females in the within-pairs model. For males, the finding of a significant relationship between the ratio and any NDD or ADHD diagnosis in the between-pairs model may suggest the influence of genetic factors in the development of the ratio, resulting in a lower 2D:4D ratio for those males with a NDD in general or an ADHD diagnosis in particular. For females, the relationship between the ratio and NDDs may be masked by confounding factors between twin pairs, such as genetics and shared environment, and therefore, the link is only observed when controlling for these factors as occurs in the within-pairs model. In contrast to published meta-analyses, which reported ratios lowered between .011 and .077 in individuals with ASD versus those without (Honekopp 2012; Teatero and Netley 2013), we did not find a relationship between the ratio and a diagnosis of ASD, consistent with results from a large recent study (Guyatt et al. 2015).

Descriptively, male pairs concordant for ASD had the lowest overall 2D:4D ratio compared with females with concordant ASD. These results are consistent with Guyatt et al. (2015), who demonstrated males with ASD to have lower overall 2D:4D ratios compared with females with ASD. In contrast to our hypotheses, the between-pairs model demonstrated a positive association between the ratio and autistic traits for females only, indicating that as the ratio increased, there was a tendency for autistic traits to also increase in females. It is unclear why this finding occurred and the results need to be interpreted with caution.

This study had several strengths, including the use of two, blind raters conducting the digit measurements and a sample of both and female twins with well-characterized NDDs and twins with TD. The twin design adjusts for genetic and environmental factors shared between twins in pairs, thereby excluding confounders like genetics. Potential limitations to this study include participants coming from a limited geographical region, the subjectivity of the measurements, even with a digital measurement system, and the broad age range of included twins for some analyses. The limited geographical region may have resulted in measurements that were more characteristic of a Northern European population as our digit ratios were much higher than what has been reported in previous studies with samples from other regions. For example, our study found that individuals with a concordant diagnosis of ASD had an overall hand 2D:4D ratio that was *Md* = 1.014 for females and *Md* = .970 for males. In contrast, Guyatt et al. (2015) studied a population with ASD in the United Kingdom and found mean ratios of 0.969 and 0.959 for females and males, respectively. Our study used a digital measurement system (i.e., Image J) where two raters demonstrated high interrater reliability for ratio assessment. Final limitations are the generalizability of study findings to populations that do not include twins, as well as potential for chance findings due to the relatively large number of statistical tests performed. Although we present some significant findings for males and females in regards to lower digit ratios and the presence of any NDDs, and ADHD specifically for males, we might have missed associations with other single NDD diagnoses, potentially owing to smaller sample sizes in the NDD subgroups.

Influential autism research paradigms like the extreme male brain theory have purported the 2D:4D ratio as a potential avenue to explore in terms of ASD etiology. The primary findings from this study, which included participants with not only ASD, but also other NDDs, suggest that the effect of testosterone on the diagnoses of ASD may not be as strong as previously thought or perhaps rather affects neurodevelopment in a broader fashion. While associations for NDD subgroups other than ADHD, specifically for ASD, might have been missed due to subgroup sample size, other minor physical features might be more closely related to altered neurodevelopment in general and ASD in particular (Myers et al. 2017) and may warrant further study.

## Electronic supplementary material

Below is the link to the electronic supplementary material.

Supplementary material 1 (DOCX 15 KB)

Histograms demonstrating distribution of mean 2D:4D hand ratio for a) all participants (n=238), b) females only (n=106), c) males only (n=132) (DOCX 3849 KB)
