# Supplementary figures and images for: 2D:4D Ratio in Neurodevelopmental Disorders: A Twin Study
Source: J Autism Dev Disord. 2018 Apr 27;48(9):3244–52. doi: 10.1007/s10803-018-3588-8 (PMC6096789; doi:10.1007/s10803-018-3588-8)

Supplemental Figure I: Histograms of Mean 2D:4D Ratio

A.


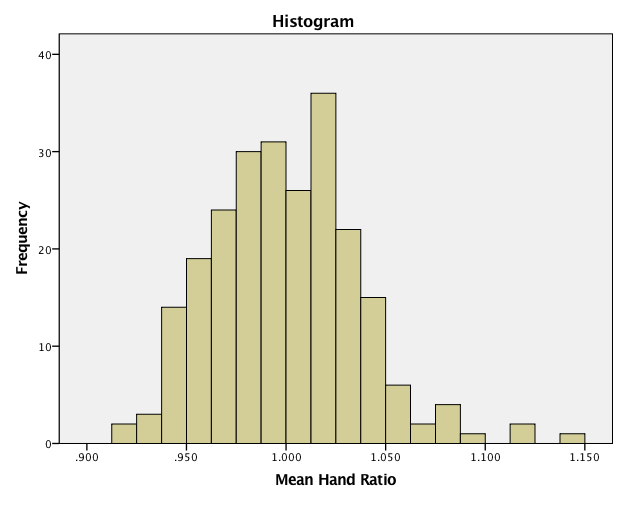


B.


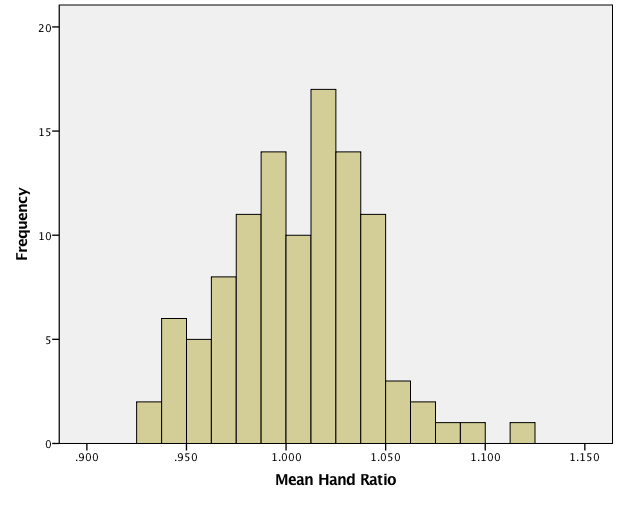


C.


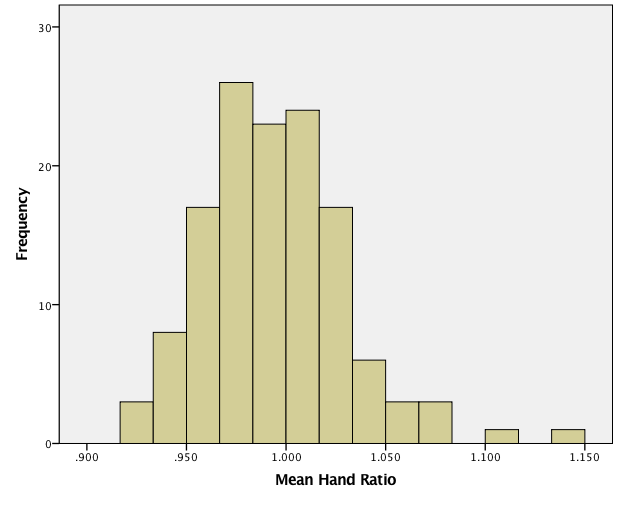

Supplement: Supplementary file 2 — Histograms demonstrating distribution of mean 2D:4D hand ratio for a) all participants (n=238), b) females only (n=106), c) males only (n=132) (DOCX 3849 KB) [file 10803_2018_3588_MOESM2_ESM.docx]
